# Feature Selection and Feature Stability Measurement Method for High-Dimensional Small Sample Data Based on Big Data Technology

**DOI:** 10.1155/2021/3597051

**Published:** 2021-09-23

**Authors:** Chengyuan Huang

**Affiliations:** School of Electricity and New Energy, China Three Gorges University, Yichang 443002, China

## Abstract

With the rapid development of artificial intelligence in recent years, the research on image processing, text mining, and genome informatics has gradually deepened, and the mining of large-scale databases has begun to receive more and more attention. The objects of data mining have also become more complex, and the data dimensions of mining objects have become higher and higher. Compared with the ultra-high data dimensions, the number of samples available for analysis is too small, resulting in the production of high-dimensional small sample data. High-dimensional small sample data will bring serious dimensional disasters to the mining process. Through feature selection, redundancy and noise features in high-dimensional small sample data can be effectively eliminated, avoiding dimensional disasters and improving the actual efficiency of mining algorithms. However, the existing feature selection methods emphasize the classification or clustering performance of the feature selection results and ignore the stability of the feature selection results, which will lead to unstable feature selection results, and it is difficult to obtain real and understandable features. Based on the traditional feature selection method, this paper proposes an ensemble feature selection method, Random Bits Forest Recursive Clustering Eliminate (RBF-RCE) feature selection method, combined with multiple sets of basic classifiers to carry out parallel learning and screen out the best feature classification results, optimizes the classification performance of traditional feature selection methods, and can also improve the stability of feature selection. Then, this paper analyzes the reasons for the instability of feature selection and introduces a feature selection stability measurement method, the Intersection Measurement (IM), to evaluate whether the feature selection process is stable. The effectiveness of the proposed method is verified by experiments on several groups of high-dimensional small sample data sets.

## 1. Introduction

At present, the research on data mining has always been a hot issue in the fields of artificial intelligence, machine learning, and database. The reason why data mining is so valued is that it can extract hidden and unknowable potential value information from a large number of complex data in the database to assist in decision-making. With the continuous emergence of large-scale data mining tasks, such as microarray gene expression data [1], which contains tens of thousands of gene features while the number of samples is small, the data dimension of the mining object is significantly expanded and the difficulty of mining is increased. With the development of big data in the future, more and more data mining tasks with high-dimensional and small sample characteristics will continue to emerge. How to process these data will also become a research difficulty: on the one hand, high data dimensionality will lead to dimensionality disasters; on the other hand, because the number of samples is too small, overfitting problems will be caused. Both will seriously reduce the classification or clustering accuracy and greatly increase the burden of learning. Therefore, in order to process high-dimensional small sample data and extract the required information from it, feature selection becomes a feasible way.

Feature selection is to filter the feature subset from the original feature space, which can effectively reduce the dimension of the feature space [2]. Feature selection does not change the original feature space structure but only selects some important features from the original features to reconstruct a low-dimensional feature space with the same spatial structure as the original feature. It is an optimization process [3]. Many existing studies have explained the significance and importance of feature selection [4–6]. At present, the mainstream feature selection methods are mainly divided into three types, namely, Filter, Wrapper, and Embedded.

Filter measures the feature classification ability by analyzing the internal features of the feature subset and is generally used to filter out the feature subset with the highest score. According to the selection of selected subsets, Filter can be divided into two types: based on feature sorting [7] and feature space search [8] such as correlation-based feature selection (CFS) [9], maximum relevance minimum redundancy (MRMR) [10], and Bayesian framework [11–13]. However, the two methods of Filter have the problem of difficulty in coordination of computational complexity and classification accuracy, which leads to unsatisfactory processing results.

As for Wrapper, it can be divided into two types: sequential search method [14] and heuristic search [15]. The sequential search strategy reduces the computational complexity by continuously adding (deleting) a single feature, but it is easy to select feature subsets whose inner features are highly correlated [16]. The heuristic search algorithm is represented by the particle swarm optimization algorithm [17]. The initial feature subset is randomly generated, and the heuristic rule is gradually approached to the optimal solution, which can meet most of the needs. However, the high cost of reconstructing the classification model when dealing with different data sets limits its further development.

The emergence of Embedded is to solve the high cost of reconstructing the classification model when Wrapper processes different data sets. Taking the SVM-RFE method proposed by Guyon et al. [18] based on the idea of recursive feature search and elimination as an example, each dimension of the SVM hyperplane corresponds to each feature in the high-dimensional small sample data set, the importance of each feature is measured by feature weight, and the lower ranked feature is deleted in descending order. The high-dimensional data dimensionality reduction work is completed after iteration, which effectively improves the time and space performance of the method and ensures high-precision classification results.

Although there are many mature feature selection methods, these methods emphasize the high classification performance or clustering performance of the feature selection results and ignore the stability of the feature selection results. The stability of feature selection refers to the insensitivity of feature selection results to small fluctuations in training content. In some situations, when the sample content changes slightly, the feature subsets or the feature importance ranking results obtained by feature selections are quite different, and even some incomprehensible feature sequences are output, which seriously reduces the accuracy of the feature selection method. This is the performance of poor feature selection stability. If the feature selection is performed by combining multiple learners in an ensemble way and the best feature selection result is selected from many learners, the stability of the feature selection result can be effectively improved. Li et al. [19] generated test objects by resampling technology and repeatedly used recursive decision trees for feature selection. Dutkowski and Gambin [20] used different feature selection algorithms for gene selection and integrated the results of each algorithm through optimization strategies to form the final feature subset. Saeys et al. [21] and Abeel et al. [22] used the bagging idea for ensemble feature selection and achieved good processing results.

Based on the above research, this paper proposes a random bits forest [23] recursive clustering elimination (RBF-RCE) feature selection method based on the idea of ensemble. First, through K-means clustering, the research object is divided into several feature clusters, random bits forest (RBF) is used to calculate the importance of any feature in the cluster, and the feature score is calculated according to the importance of the feature. Then, after sorting in descending order according to the feature scores, the relevant deletion parameters are set. By judging the relationship between the number of existing features and the deletion parameters, the features in the cluster are deleted in reverse order to achieve feature dimensionality reduction processing. In addition, by analyzing the reasons for the unstable feature selection, this paper introduces a feature selection stability measurement method, which measures whether the feature selection is stable or not through the intersection measurement (IM). Eventually, through experiments on high-dimensional and small-sample data sets, the results demonstrate the effectiveness of the method and can achieve highly stable feature selection results.

## 2. RBF-RCE Feature Selection Method

Random forest has many advantages when dealing with high-dimensional small sample data [24], while random bits forest is improved by random forest, and it performs better on classification problems. In this paper, based on the random bits forest, combined with the support vector machine-based recursive clustering elimination feature selection method proposed by Yousef et al. [25] and the improved SVM-RCE method proposed by Luo et al. [26], a random bits forest recursive clustering elimination feature selection method is proposed. The following is a detailed description of the overall approach.

### 2.1. Feature Importance Analysis Based on Random Bits Forest

**Figure 1:**
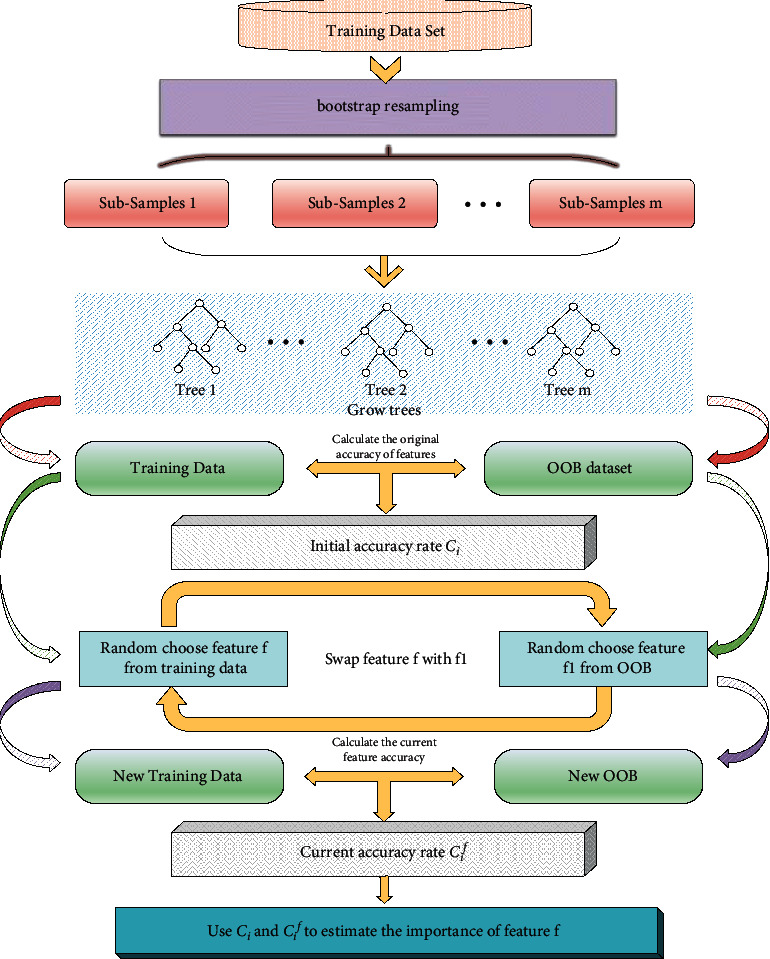
Feature importance calculation based on random bits forest OOB replacement.

Random bits forest has been applied to high-dimensional small sample data processing due to its good performance in data classification processing. It inherits the characteristics of random forest screening by the importance of each feature when performing feature selection and combines neural network [27] to improve model depth, gradient boosting [28, 29] extends model breadth, and random forest [24] improves model classification accuracy. In dealing with the problem of high-dimensional small sample data, it has higher accuracy and algorithm convergence than random forest. For a high-dimensional small sample data set, random bits forest uses Bootstrap resampling technology [30], random sampling with replacement *N* times to obtain *M* sample sets, about 36% of the original samples have not been sampled, this part of the data is classified as out-of-bag (OOB) data, and the importance of features is evaluated through out-of-bag data; the process is shown in Figure 1.

Specific steps are as follows:

(1) Based on the high-dimensional small sample data set *Z*={(*x*_1_, *y*_1_),…, (*x*_*n*_, *y*_*n*_)}, a random bits forest model is established, the OOB data set of the *i*thtree as *I*_*i*_^OOB^ is set, and the OOB accuracy rate is *C*_*i*_.
(2) Any feature *f* from the data set *Z* is taken, the features in OOB are selected randomly to replace, a new data set *Z*_1_ is obtained, the OOB accuracy rate *C*_*i*_^*f*^ of the *i*th tree is recalculated, and the difference in OOB accuracy before and after feature replacement is obtained:
  (1) $$\begin{array}{c} E_{i}^{f}=C_{i}-C_{i}^{f}, \end{array}$$
where *i*=1,2,…, *m*.
(3) According to the difference of accuracy rate, the influence degree of feature *f* on OOB accuracy rate can be obtained:
  (2) $$\begin{array}{c} E^{f}=\frac{\sum_{i=1}^{m}E_{i}^{f}}{m}. \end{array}$$
(4) The variance of *E*^*f*^ is as follows:
  (3) $$\begin{array}{c} S^{2}=\frac{\sum_{i=1}^{m}{\left(E_{i}^{f}-E^{f}\right)}^{2}}{m-1}. \end{array}$$
(5) The importance of feature *f* can be inferred as follows:
  (4) $$\begin{array}{c} f_{ip}=\frac{E^{f}}{S}. \end{array}$$

So far, the importance of each feature in the high-dimensional small sample data set is calculated by random bits forest. However, how to further implement feature selection based on the known importance of each feature is still a problem. Above this, the recursive clustering elimination idea is introduced to realize the screening of feature subsets.

### 2.2. Recursive Clustering Elimination Idea

The idea of recursive clustering is to cluster the original features into several feature classes and then combine the algorithm to score each feature class, eliminate low-scoring feature classes, and iterate until the initial set termination conditions are met, to obtain the final feature subset. The introduction of recursive clustering ideas can effectively improve the efficiency of random bits forests in feature selection, improve the classification accuracy of feature selection, and achieve rapid convergence of the algorithm.

In the feature clustering stage, the K-means clustering algorithm [31] is used to divide the features into different feature classes by the distance between each feature for subsequent feature selection. The formula for calculating the characteristic distance is as follows [32]:

(5) $$\begin{array}{c} D_{ij}=1-\frac{\sum_{r=1}^{n}\left(f_{ir}-{\bar{f}}_{i}\right)\left(f_{jr}-f_{j}\right)}{\sqrt{\sum_{r=1}^{n}{\left(f_{ir}-{\bar{f}}_{i}\right)}^{2}}\sqrt{\sum_{r=1}^{n}{\left(f_{jr}-{\bar{f}}_{j}\right)}^{2}}}. \end{array}$$

Among them, ${\bar{f}}_{i}=\left(\sum_{r=1}^{n}f_{ir}/n\right)$ and *f*_*ir*_ represents the *r*th feature of cluster *i*th and *j*th.

Through the K-means clustering algorithm, the initial features can be clustered into *n* feature clusters. After the feature clustering is completed, based on the random bits forest feature importance calculation, a feature class score function Score(*S*_*i*_) is defined. The specific formula for calculation is as follows:

(6) $$\begin{array}{c} \text{Score}\left(S_{i}\right)=\underset{j\in S_{i}}{\max}\left|f_{ip}^{j}\right|. \end{array}$$

In the formula, *f*_*ip*_^*j*^ refers the importance of *i*th feature in feature class *S*(*i*). The specific calculation method refers to formulas (1)–(4).

**Figure 2:**
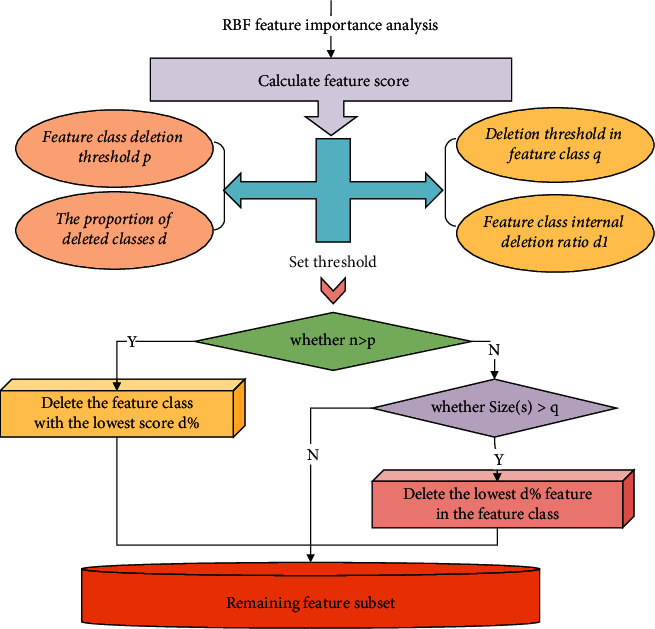
Recursive deletion process.

After the feature scores of each feature class are obtained through the above formula, recursive deletion can be performed according to the feature scores. Because each recursion deletes a certain proportion of feature classes, irrelevant features can be quickly filtered out during the initial stage of feature selection. However, once faced with a situation where the number of features is small, further feature deletion will delete important features in the feature class. Therefore, by setting a series of deletion condition thresholds, once the number of feature classes is less than the set threshold, the operation of deleting feature classes will be transformed into deleting features in the feature classes. In this way, redundant features can be quickly eliminated in the early stage of feature selection, and important features can be effectively identified and screened in the later stage of feature selection. The overall deletion idea is shown in Figure 2.

**Figure 3:**
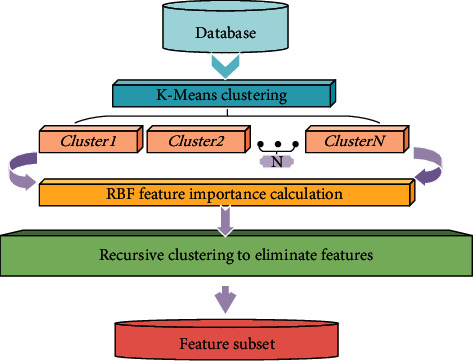
RBF-RCE feature selection method process.

Through the combination of RBF and recursive clustering elimination ideas, the overall RBF-RCE feature selection processing flow is shown in Figure 3.

## 3. A New Stability Measurement Method for Feature Selection

The stability of feature selection refers to the insensitivity of feature selection results to changes in the training set. Highly stable feature selection results can ensure that even when the research object undergoes small fluctuations, the resulting feature selection results will not change significantly or even output results that deviate from the real situation. In recent years, with the in-depth study of feature selection methods, how to maintain the performance of feature selection classification while improving the stability of feature selection has begun to receive more and more attention. This paper analyzes the causes of unstable feature selection and introduces a new measurement method of feature selection stability to evaluate whether the feature selection method is stable.

### 3.1. Reasons for Unstable Feature Selection

Through the query of the relevant literature and the in-depth analysis of various feature selection algorithms, it can be concluded that there are three main reasons for the instability of feature selection: the feature selection algorithm itself, the number of selected objects, and the data attributes.

For the feature selection algorithm itself, since most of the current feature selection algorithms mainly consider improving classification performance or clustering performance when designing evaluation criteria, they do not fully consider the stability of feature selection, resulting in poor stability of feature selection results.

The number of selected objects directly affects the stability of the feature selection results. Since the number of features K in the optimal feature subset cannot be known in advance, the specific number of selections is generally set manually. Through research, it is found that the larger the number of selected features, the higher the stability of feature selection. The main reasons are as follows:

1. An increase in *K* will increase the probability of all relevant features being selected
2. The increase in *K* will increase the number of elements in the intersection between the selected feature subsets

If *f*_*i*_ and *f*_*j*_ are feature subsets obtained after feature selection by the same feature selection algorithm on the training set *D*_*i*_ and *D*_*j*_, the prior probability of randomly selecting a feature *f* is as follows [33]:

(7) $$\begin{array}{c} p\left(f\right)=\frac{1}{m}. \end{array}$$

The probability of selecting *K* features is as follows:

(8) $$\begin{array}{c} p\left(f_{i}\right)=p\left(f_{j}\right)=\frac{1}{\left(\begin{array}{c} m\\ k \end{array}\right)}. \end{array}$$

The probability that *f*_*i*_ and *f*_*j*_ have at least one same feature is as follows:

(9) $$\begin{array}{c} p\left(\left|f_{i}\cap f_{j}\right|\geq 1\right)=\frac{\left(\begin{array}{c} m\\ m-k \end{array}\right)}{\left(\begin{array}{c} m\\ k \end{array}\right)}. \end{array}$$

By the above equation, the larger *k* is, the higher *p*(|*f*_*i*_∩*f*_*j*_| ≥ 1) is, which means the higher feature selection stability.

Data attributes will also affect the stability of feature selection. Data attributes can be subdivided into data dimensions *D*, the number of data samples N, and the degree of feature redundancy in the data. For feature dimensions, when the number of features is determined, the larger the data dimension *D*, the smaller the value of *p*(*f*_*i*_=*f*_*j*_). *p*(*f*_*i*_=*f*_*j*_) represents the prior probability of the same feature in *f*_*i*_ and *f*_*j*_, which can be known in combination with the following formula:

(10) $$\begin{array}{c} p\left(f_{i}=f_{j}\right)=\frac{1}{\left(\begin{array}{c} m\\ k \end{array}\right)}=\frac{k!\left(m-k\right)!}{m!}. \end{array}$$

Combined with the research of literature [34], it is pointed out that limited training samples will increase the degree of overfitting and reduce the performance of learning generalization.

Finally, the feature selection result will generate multiple feature subsets with similar performance due to the high redundancy of the features of the sample set, which also affects the stability of feature selection.

Combined with the above analysis, the stability of feature selection results will be interfered by many factors. In order to measure the stability of feature selection results, this paper introduces a new feature selection stability measurement method for judgment.

### 3.2. Evaluation of Feature Selection Stability Based on Intersection Metric

This paper introduces a new feature selection stability measurement method-Intersection Metric (IM) to measure the stability of feature selection.

The intersection measurement was originally proposed by Fagin et al. [35]. It is a measurement method based on the combination of feature ranking and feature subsets. It is commonly used to define the distance between two Top*k* lists. Combined with standardized European distance [36], the distance is measured from the two feature lists. The greater the distance, the smaller the similarity. If *f*_*i*_ and *f*_*j*_ are the ranking vectors obtained by feature selection of the same feature selection algorithm on the training set *D*_*i*_ and *D*_*j*_, then the IM between *f*_*i*_ and *f*_*j*_ is as follows [35]:

(11) $$\begin{array}{c} \text{IM}\left(f_{i},f_{j}\right)=\frac{\sum_{i=1}^{k}\delta_{t}\left(f_{i},f_{j}\right)}{k}, \end{array}$$

(12) $$\begin{array}{c} \delta_{t}=\left(f_{i},f_{j}\right)=\frac{\left|f_{i}^{t}/f_{j}^{t}\right|\cup \left|f_{j}^{t}/f_{i}^{t}\right|}{2t}. \end{array}$$

Among them, (*f*_*i*_^*t*^/*f*_*j*_^*t*^) represents the relative difference set of vector *f*_*i*_ to vector *f*_*j*_, *k*=|*f*_*i*_|=|*f*_*j*_|, and *f*_*i*_^*t*^  is the first *t* part of *f*_*i*_.

According to the above IM calculation method, the definition of similarity measure in this article is as follows:

(13) $$\begin{array}{c} {\text{sim}}_{\text{IM}}\left(f_{i},f_{j}\right)=1-\text{IM}\left(f_{i},f_{j}\right). \end{array}$$

By introducing formula 11, formula (13) can be transformed into the following:

(14) $$\begin{array}{c} {\text{sim}}_{\text{IM}}\left(f_{i},f_{j}\right)=1-\frac{\sum_{t=1}^{k}\delta_{t}\left(f_{i},f_{j}\right)}{k}. \end{array}$$

The value range of the intersection metric sim_IM_(*f*_*i*_, *f*_*j*_) is [0, 1]. When the value approaches 1, it indicates that the similarity between *f*_*i*_ and *f*_*j*_ is greater.

The intersection measurement method proposed in this paper can measure the stability of feature selection by measuring the similarity between the rankings in the feature ranking vector and realizing the stability evaluation of the feature selection results.

## 4. Simulation Results and Discussion

### 4.1. Evaluation of Random Bits Forest Recursive Clustering Elimination Method

In order to verify the effectiveness of the feature selection method proposed in this paper, experiments are carried out with the following groups of high-dimensional small sample data sets. The specific description of the data set is shown in Table 1, and the corresponding references detail the source of the data. The traditional random forest algorithm, SVM-RCE algorithm, and random bits forest recursive clustering elimination (RBF-RFE) algorithm are combined to evaluate the time efficiency and classification accuracy. Through the feature selection stability evaluation method proposed in this paper, the feature selection stability of the three methods is analyzed and compared.

In order to ensure the stability of the experimental results, this paper uses 2/3 of the data set as the training set and the remaining 1/3 as the test set. Repeat 10 times to average the results, and the data sets are all preprocessed. After 10 feature selections, the average of the time consumed by the three algorithms is counted. The results are shown in Table 2.

In terms of time efficiency, for the data sets Colon, DLBCL, and Prostate, RBF-RCE consumes much less time than the other two methods; for the ultra-high-dimensional data set GLI, SVM-RCE exceeds the memory during calculation, and the results cannot be obtained. RBF-RCE takes about 1/3 of the time consumed by RF, indicating that RBF-RCE is more time efficient than SVM-RCE and RF when processing high-dimensional small sample data or even ultra-high-dimensional data and can solve the feature selection problem of ultra-high-dimensional data sets.

**Figure 4:**
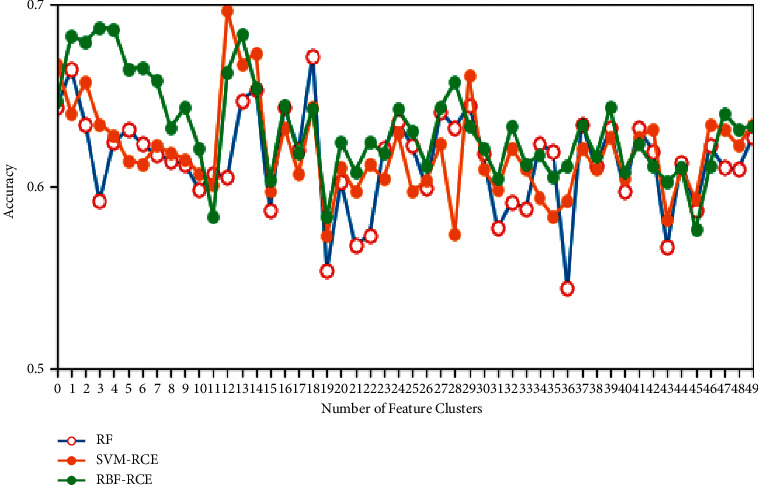
Classification accuracy on the Colon dataset.

**Figure 5:**
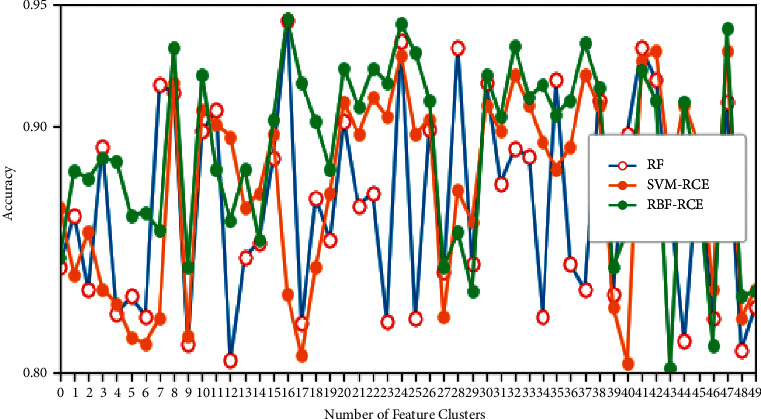
Classification accuracy on the DBLCL dataset.

**Figure 6:**
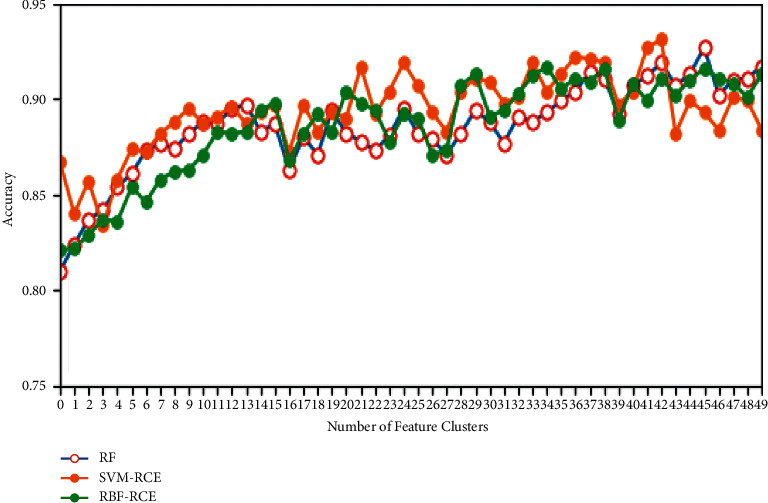
Classification accuracy on the Prostate dataset.

**Figure 7:**
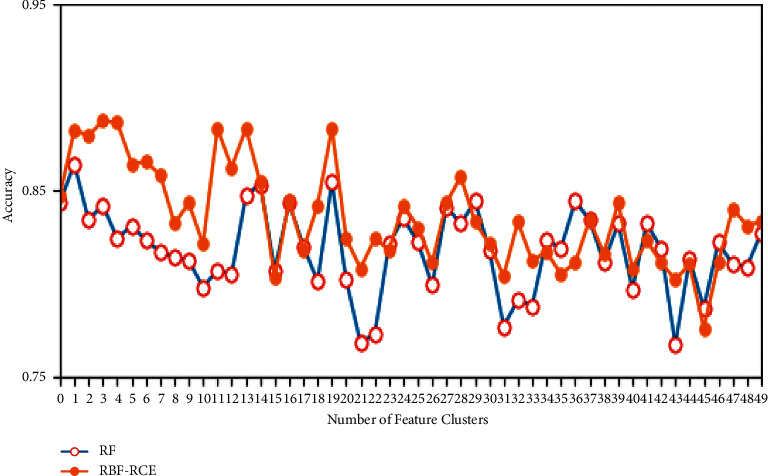
Classification accuracy on the GLI dataset.

In terms of classification accuracy, the classification of the three algorithms on different data sets is plotted as shown in Figures 4–7. It can be seen from the figure that for the data sets Colon, DLBCL, and Prostate, the classification accuracy of the three algorithms is not much different. On the Colon dataset, the classification accuracy of the three algorithms is 61.39% (RF), 61.89% (SVM-RCE), and 63.13% (RBF-RCE). The classification accuracy of the RBF-RCE algorithm is slightly higher. On the DBLCL dataset, the classification accuracy is 86.80% (RF), 87.49% (SVM-RCE), and 88.95% (RBF-RCE). Similarly, the RBF-RCE feature selection method performs better in terms of classification accuracy. On the Prostate dataset, the classification accuracy is 88.56% (RF), 89.38% (SVM-RCE), and 88.62% (RBF-RCE). The classification performance of RBF-RCE is slightly inferior to SVM-RCE, with an accuracy difference of only 0.76%, but it is still stronger than traditional RF feature selection methods. For the performance on the ultra-high-dimensional data set GLI, since the SVM-RCE algorithm cannot obtain the feature selection results, only the classification performance of the RF and RBF-RCE algorithms is analyzed. The classification accuracy of the two is 81.79% (RF) and 83.53% (RBF-RCE). When dealing with ultra-high-dimensional data, the RBF-RCE feature selection method can still achieve good classification performance.

Based on the classification accuracy of the above three algorithms in different data sets, perhaps because the objective data sets will produce differences in classification performance, the overall RBF-RCE can achieve good results in the classification of high-dimensional and small-sample data sets.

After the classification accuracy is considered, the feature selection stability evaluation method based on the intersection metric mentioned in this article is used to determine the feature selection stability of the three methods. Because the feature selection process of RF and SVM-RCE is stable, RF adopts an ensemble method to combine multiple decision trees to improve stability, while the high stability of SVM-RCE is due to the introduction of recursive clustering elimination ideas. Therefore, these two methods are used as references to evaluate the stability of RBF-RCE feature selection.

**Figure 8:**
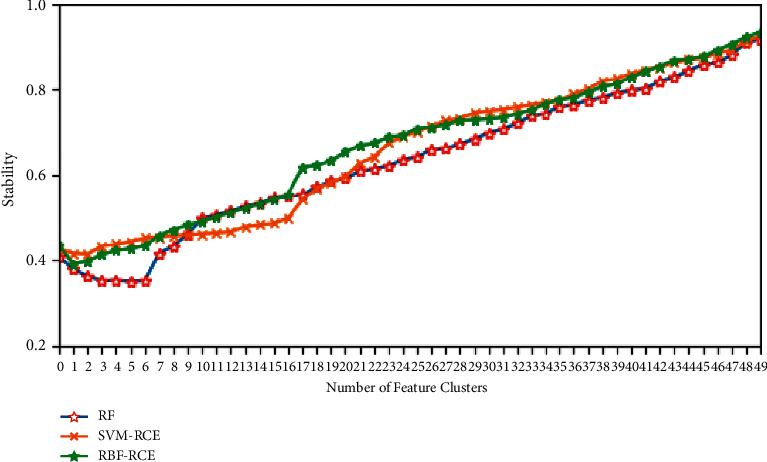
Stability evaluation on Colon data set.

**Figure 9:**
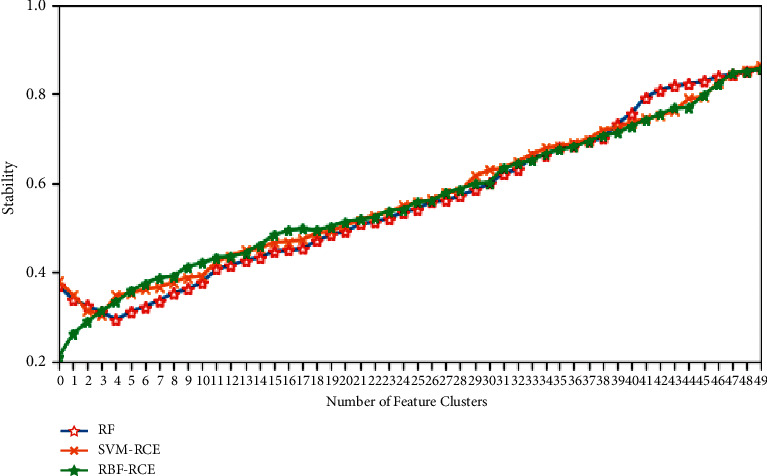
Stability evaluation on DBLCL data set.

**Figure 10:**
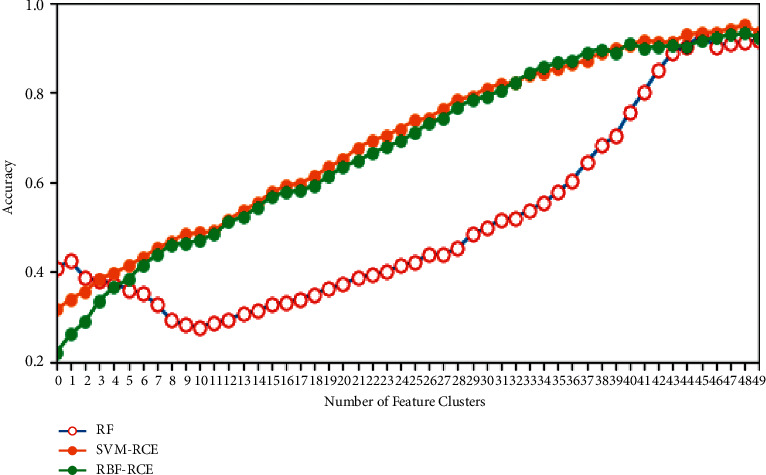
Stability evaluation on Prostate data set.

**Figure 11:**
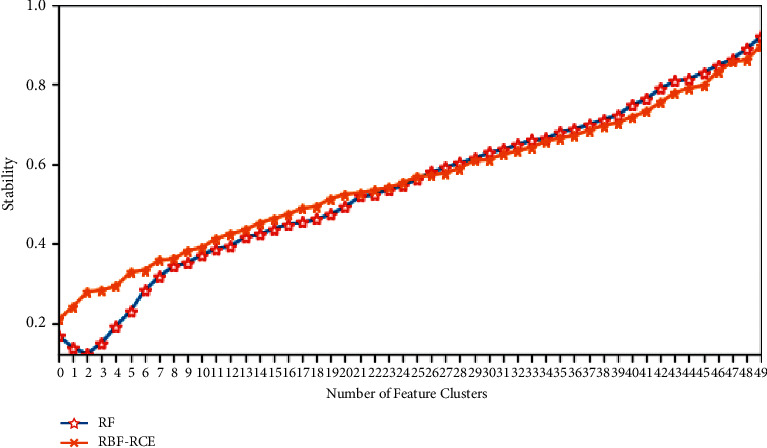
Stability evaluation on GLI data set.

The specific stability evaluation results are shown in Figures 8–11. It can be found from the figure that except for the GLI ultra-high-dimensional data set, the three methods may be different in the initial stability due to the algorithm logic, but in the end, they can reach very close feature stability, and no matter what data set is based on, the stability of feature selection always increases with the increase in the number of features. In terms of the feature selection stability of the ultra-high-dimensional data set GLI, RBF-RCE and RF can finally reach almost the same level of stability, which can show that the RBF-RCE feature selection method mentioned in this article has high feature selection stability.

### 4.2. Discussion

Based on the above analysis, some conclusions can be drawn. When dealing with high-dimensional and small-sample data sets, such as Colon and DBLCL, when the data dimension is higher than the sample size or in the context of dealing with ultra-high-dimensional data sets, such as GLI, the RBF-RCE feature selection method can eliminate irrelevant feature classes and achieve rapid reduction of feature dimensions, so it can achieve excellent performance in time efficiency. It also optimizes the classification performance of traditional feature selection methods, overcomes the shortcomings of SVM-RCE that cannot process ultra-high-dimensional data, and improves the classification accuracy of traditional RF feature selection methods. Through the feature selection stability measurement method based on intersection metric proposed in this paper, the stability evaluation of several feature selection methods is carried out. The results show that RBF-RCE can finally achieve almost the same stability as traditional feature selection methods on different data sets. It shows that the RBF-RCE feature selection method not only has excellent time efficiency and classification accuracy but also can achieve better feature selection stability and can be used to process high-dimensional small sample data sets. In addition, more research can be carried out in the future based on the uncertainty of actual data. And a fuzzy clustering-based approach [40] is a good solution that is worth conducting more in-depth research in the future.

## 5. Conclusion

In summary, this article combines the feature importance analysis of random bits forests, introduces the idea of recursive clustering elimination, and proposes a new high-dimensional small sample data feature selection method RBF-RCE, which performs better than traditional feature selection methods in terms of time efficiency and classification accuracy. This is also confirmed by tests on actual data sets. On this basis, this article further proposes a feature selection stability measurement method to evaluate the stability of feature selection for many feature selection methods, combined with the intersection measurement, which will help to measure the reliability of the feature selection method whether it can obtain a true and interpretable feature subset that meets actual needs. The research content of this article can provide a new and effective method for the processing of high-dimensional small sample data and can provide a reliable solution for the majority of researchers when facing high-dimensional or even ultra-high-dimensional data sets.

## Figures and Tables

**Table 1:** High-dimensional small sample data set.

Data set	Feature number	Number of samples	Source
Colon	2000	62	[36]
GLI	22283	85	[37]
DLBCL	5469	77	[38]
Prostate	6033	102	[39]

**Table 2:** The time consumed by the three algorithms (s).

Data set	RBF-RCE	SVM-RCE	RF
Colon	**15.23**	30.29	27.24
GLI	**945.38**	None	2874.52
DLBCL	**124.25**	377.56	207.43
Prostate	**179.33**	665.81	371.64

The bold value indicates that the algorithm is most efficient, and the shortest time consumption is handled in the same data set.

## Data Availability

Previously reported data were used to support this study and are available at 10.1073/pnas.96.12.6745, 10.1016/j.ijmedinf.2005.05.002 and 10.1016/S1535-6108(02)00030-2. These prior studies (and datasets) are cited at relevant places within the text as references [36–39].
